# Biological implications and clinical potential of invasion and migration related miRNAs in glioma

**DOI:** 10.3389/fnint.2022.989029

**Published:** 2022-11-21

**Authors:** Xin Guo, Hengxing Jiao, Lele Cao, Facai Meng

**Affiliations:** Department of Neurosurgery, Shaanxi Provincial People's Hospital, Xi'an, China

**Keywords:** glioma, miRNAs, migration and invasion, biological implications, clinical potential

## Abstract

Gliomas are the most common primary malignant brain tumors and are highly aggressive. Invasion and migration are the main causes of poor prognosis and treatment resistance in gliomas. As migration and invasion occur, patient survival and prognosis decline dramatically. MicroRNAs (miRNAs) are small, non-coding 21–23 nucleotides involved in regulating the malignant phenotype of gliomas, including migration and invasion. Numerous studies have demonstrated the mechanism and function of some miRNAs in glioma migration and invasion. However, the biological and clinical significance (including diagnosis, prognosis, and targeted therapy) of glioma migration and invasion-related miRNAs have not been systematically discussed. This paper reviews the progress of miRNAs-mediated migration and invasion studies in glioma and discusses the clinical value of migration and invasion-related miRNAs as potential biomarkers or targeted therapies for glioma. In addition, these findings are expected to translate into future directions and challenges for clinical applications. Although many biomarkers and their biological roles in glioma invasion and migration have been identified, none have been specific so far, and further exploration of clinical treatment is still in progress; therefore, we aimed to further identify specific markers that may guide clinical treatment and improve the quality of patient survival.

## Introduction

Glioma is the most common primary central malignancy, accounting for 80% of all malignant tumors of the brain (Siegel et al., 2015; Torre et al., 2015). The WHO classifies gliomas into low-grade groups (grades I and II) and high-grade groups (grades III and IV) (Huo et al., 2020). Fatal aggressiveness and a high recurrence rate are among its malignant features (Katakowski et al., 2010; Zhou et al., 2013). Despite the increasing sophistication of conventional treatments such as surgical resection and radiotherapy (de Groot et al., 2010), the prognosis is often extremely poor due to its malignant behavior, as well (Parker et al., 2013). Therefore, elucidation of the mechanisms of glioma progression is urgently needed to improve clinical outcomes.

High aggressiveness leads to poor prognosis and treatment resistance in patients with glioma. Clarifying the mechanisms of glioma aggressiveness will help improve patient prognosis. Important factors affecting tumor invasion and migration include Epithelial-to-mesenchymal transition (EMT), hypoxia, angiogenesis, and tumor microenvironment (TME). EMT is the process of epithelial to mesenchymal transition (Wurdinger et al., 2008). By regulating genetic programming, hypoxia allows cancer cells to acquire a more aggressive phenotype, which in turn allows body cells to adapt to the hypoxic environment (Kim et al., 2018). Angiogenesis is an important basis for the growth of solid tumors, including gliomas, and is closely related to the migration and invasion of gliomas (Folkman, 1971; Poleszczuk et al., 2015; Dai et al., 2018). Additionally, the tumors microenvironment is also crucial for tumors progression and invasion (Ren et al., 2014). Changes in cancer cell metabolism can increase tumors cell acid production, leading to normal cell death and protease hydrolase degradation of the extracellular matrix, thereby enhancing the migration and invasion ability of cancer cells (Williams et al., 1999; Lardner, 2001; Gatenby and Gawlinski, 2003). In addition, cell adhesion molecules, such as L1 cell adhesion molecule (L1CAM), can stimulate cell motility, proliferation, and invasion by interacting with two binding partners, integrins and fibroblast growth factor receptors (FGFRs) (Mohammadi et al., 1998; Bansal et al., 2003).

MicroRNAs (miRNA) are non-coding RNAs (ncRNAs) that are widely found in eukaryotes and are ~22 nucleotides in length (Takahashi et al., 2009; Su et al., 2014; Michailidi et al., 2015). Regulates cellular functions, including migration and invasion, by binding to target mRNAs (Yang et al., 2011; Bovell et al., 2013; Kumar et al., 2013; Yin et al., 2015). Several studies have shown that alterations in miRNA expression can regulate apoptosis, proliferation, tumorigenesis, invasion, and migration, and can also be involved in the occurrence, development, and recurrence of gliomas (Godlewski et al., 2008; Lin et al., 2013; Singh et al., 2013; Yeh et al., 2013; Zhang et al., 2019). In gliomas, miRNAs are involved in invasion and migration through cellular processes related to interactions with EMT, hypoxia, angiogenesis, and the TME. Additionally, miRNAs are able to exert pro- or anti-cancer effects in different signaling pathways, including classical wnt/β-catenin, epidermal growth factor receptor (EGFR), and transforming growth factor (TGF-β), but the exact details are not yet clear. It is imperative to elucidate the mechanisms of miRNAs associated with glioma invasion and migration, to clarify the biomarkers and therapeutic targets associated with glioma, and to provide a theoretical basis for clinical treatment. Therefore, in this study, we aimed to further identify specific markers that may guide clinical treatment and improve the quality of patient survival.

## miRNAs and cellular processes

### miRNAs and EMT process

**Figure 1:**
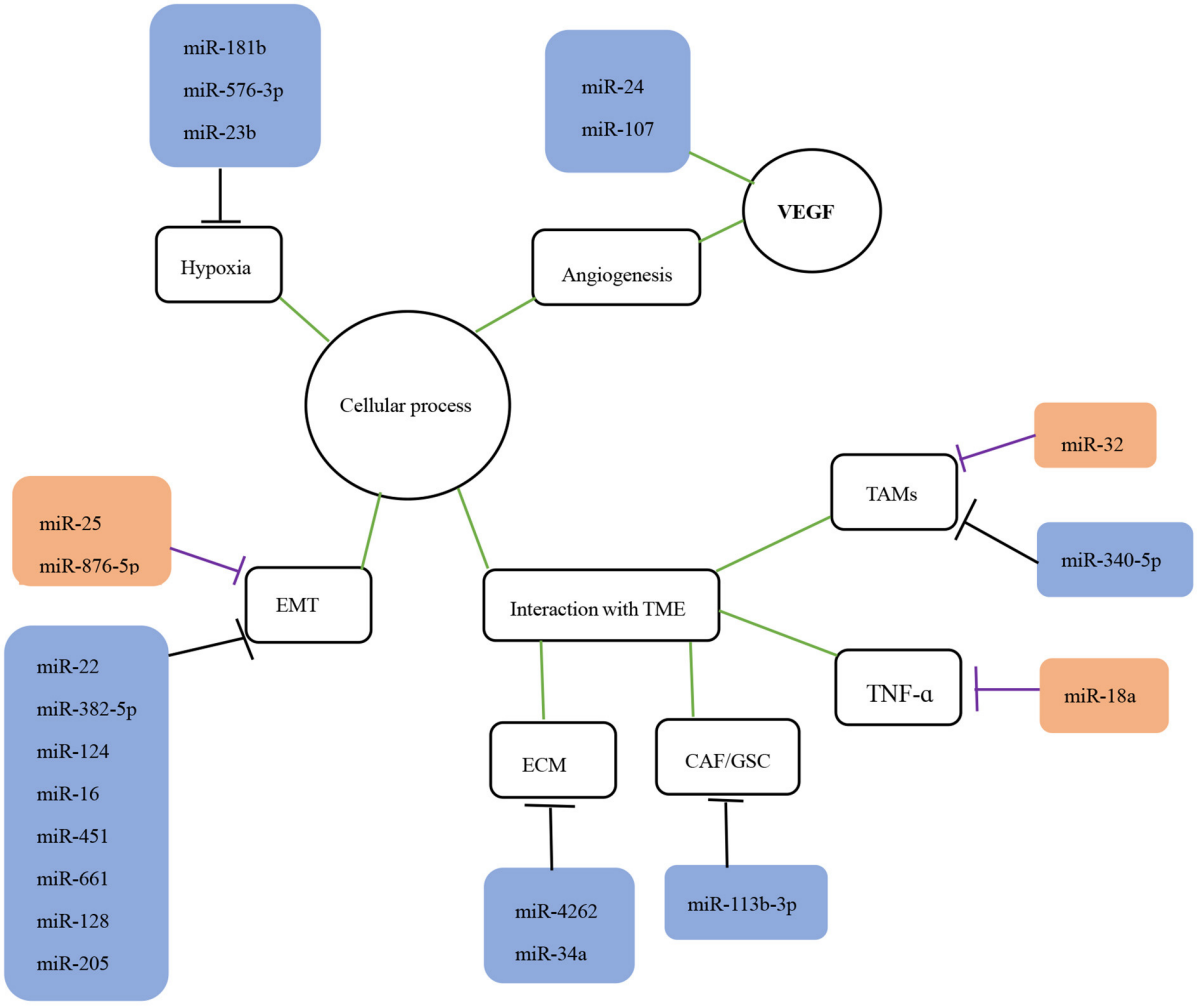
The role of miRNAs in the invasion and migration of glioma. The role of miRNAs in glioma migration and invasion is shown. The black lines indicate suppression while purple lines indicate promotion of downstream targets or processes. The green lines indicate interaction with corresponding processes or molecules. EMT, epithelial-mesenchymal transition; TME, tumor microenvironment; TAMS, tumor-associated macrophages; VEGF, vascular endothelial growth factor.

**Figure 2:**
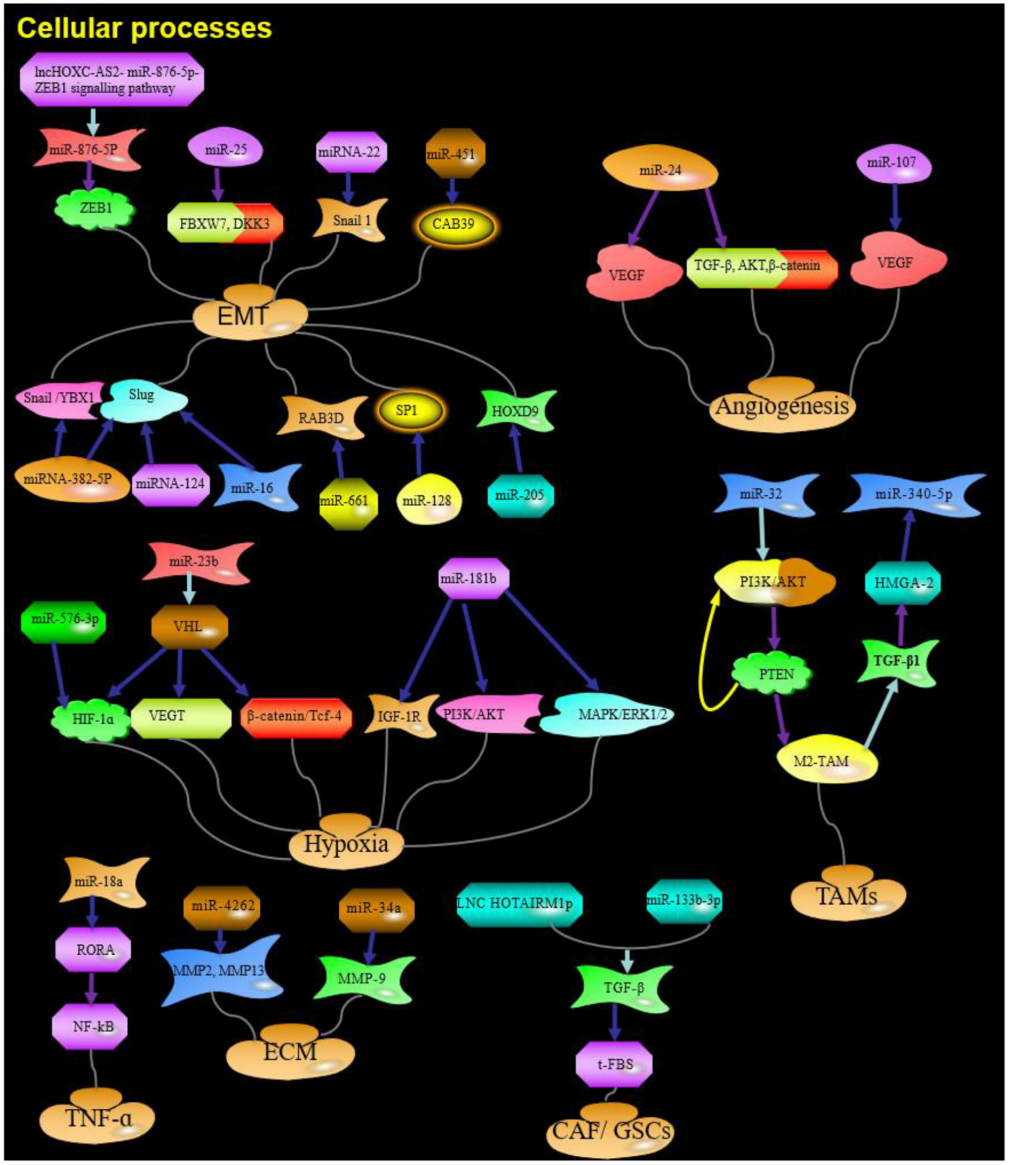
The role and targets of miRNA in cellular processes of glioma. Purple arrow indicates promotion; Blue arrows indicate suppression; Red arrows indicate positive feedback; Yellow arrows indicate negative feedback; Water green arrow indicates adjustment; ZEB1, zinc finger E-box binding homeobox 1;ZEB2, zinc finger E-box binding homeobox 2; F-box, F-box-containing; FBXW7, F-box-containing and WD repeat domain 7; DKK3, Dickkopf Wnt signaling pathway inhibitor 3; YBX1, Y box binding protein 1; HIF-1α, Hypoxia-inducible factor 1a; IGF-1R, insulin-like growth factor 1 receptor; HGS, hepatocyte growth factor-regulated tyrosine kinase substrate; TGF-β, transforming growth factor; PTEN, Phosphatase and tensin homolog; RORA, Retinoic acid receptor-related orphan receptor A; HOTAIRM1, HOXA transcript antisense RNA, myeloid-specific 1; ECM, extracellular matrix; Red and yellow arrows: Feedback.

miRNAs influence tumors development by regulating various biological processes, such as cell proliferation, apoptosis, migration, and invasion (Hu et al., 2017; Li et al., 2017). Based on the extensive miRNA literature related to glioma invasion and migration, the cellular processes known to be associated with invasion and migration can be classified into four categories: EMT, angiogenesis, hypoxia, and TME interactions (Figures 1, 2). The dysregulated miRNAs with clear targets and corresponding cellular processes are listed in Table 1.

**Table 1:** Cellular processes related to miRNA in glioma migration and invasion.

**Cellular processes**	**miRNA**	**Type of study**	**Subjects**	**Pression or knock out**	**Cell culture**	**Alteration**	**Downstream**	**References**
EMT	miR-25	Clinical	Human	Over pression	DMEM	↑	FBXW7, DKK3	Peng et al., 2019
	miR-876-5P	U-87, U-118 MG, M059K and Hs 683	Human	Low expression	DMEM	↑	ZEB1	Dong et al., 2019
	miRNA-22	Clinical	Human	Over pression	RPMI-1640 medium	↓	Snail 1	Zhang et al., 2020
	miRNA-382-5P	Clinical	Human	Over pression	DMEM	↓	Snail, Slug, YBX1	Wang J. et al., 2019
	miRNA-124	Clinical	Human	Low expression	RPMI-1640 medium	↓	Slug	Xie et al., 2012
	miR-16	Clinical	Human	Over pression	DMEM	↓	Slug	Wang Q. et al., 2014
	miR-451	Institute of Biochemistry and Cell Biology	Human	Over pression	DMEM	↓	CAB39	Nan et al., 2021a
	miR-661	Clinical	Human	Low expression	RPMI-1640 medium	↓	RAB3D	Jin et al., 2020
	miR-128	CGGA data	Human	Over pression	DMEM	↓	SP1	Dong et al., 2014
	miR-205	Clinical	Human	Over pression	DMEM	↓	HOXD9	Dai et al., 2019
Hypoxia	miR-576-3p	Clinical	Human	Over pression	DMEM	↓	HIF-1α	Hu et al., 2019
	miR-23b	Clinical	Human	Low expression	DMEM	↓	VHL, HIF-1α, VEGF	Chen et al., 2012a
	miR-181b	Clinical	Human	Over pression	DMEM	↑	IGF-1R, PI3K/AKT, MAPK/ERK1/2	Shi et al., 2013
Angiogenesis	miR-24	U251 glioma cell line and HUVECs	Human	Over pression	DMEM	↑	VEGF, TGF-β, AKT, β-catenin	Dai et al., 2018
	miR-107	U87 and A172 cell	Human	Over pression	DMEM	↓	VEGF	Chen et al., 2016
TAMs	miR-32	Human glioblastoma cell line U87 and human monocytic cell line THP1	Human	Over pression	RPMI-1640 medium	↑	PTEN	Bao and Li, 2019
	miR-340-5p	TCGA data	Human	Over pression	DMEM and RPMI-1640 medium	↓	HMGA-2	Liu Y. et al., 2019
TNF-α	miR-18a	T98G, LN229, U87, U118, U251, H4, and U178 cells	Human	Over pression	DMEM	↑	RORA, NF-KB	Jiang et al., 2020
CAF/GSCs	miR-133b-3p	Clinical	Human	Over pression	DMEM	↓	HOTAIRM1	Wang H. et al., 2021
ECM	miR-4262	NHAs, HEK-293 T, T98 G, LN18, U251, LN229 and U87	Human	Low expression	DMEM	↓	MMP2, MMP13	Yang et al., 2020
	miR-34a	Clinical	Human	Over pression	DMEM	↓	MMP-9	Wang X. et al., 2019

ZEB1, zinc finger E-box binding homeobox 1; ZEB2, zinc finger E-box binding homeobox 2; F-box, F-box-containing; FBXW7, F-box-containing and WD repeat domain 7; DKK3, Dickkopf Wnt signaling pathway inhibitor 3; YBX1, Y box binding protein 1; HIF-1α, Hypoxia-inducible factor 1a; IGF-1R, insulin-like growth factor 1 receptor; HGS, hepatocyte growth factor-regulated tyrosine kinase substrate; TGF-β, transforming growth factor; PTEN, Phosphatase and tensin homolog; RORA, Retinoic acid receptor-related orphan receptor A; HOTAIRM1, HOXA transcript antisense RNA, myeloid-specific 1; ECM, extracellular matrix; RPMI-1640, Roswell Park Memorial Institute-1640 medium; DMEM, Dulbecco's Modified Eagle Medium; NHAs, Normal human astrocytes; HEK-293 T, human embryonic kidney cell;↑, promotion;↓,suppression.

Epithelial-to-mesenchymal transition is a well-studied physiological process, based on its typical features of imbalanced expression of epithelial markers (E-cadherin) and mesenchymal markers (N-cadherin and Vimentin) leading to a more aggressive or metastatic phenotype (Yang et al., 2008; Thiery et al., 2009), which is the reason they function as glioma-promoting genes. Studies have confirmed that EMT plays an important role in the migration and invasive activity of tumor cells (Wang Q. et al., 2014; Chen et al., 2019). Its transcription factors include zinc finger E-box binding homeobox 1 (ZEB1), Snail, Slug, and Twist 1 (Wellner et al., 2009; Myung et al., 2014). E-cadherin protein expression is downregulated upon binding of ZEB1 to its promoter, resulting in glioma cell separation and migration (Edwards et al., 2011). EMT can also be induced by miRNA-related signaling pathways. miR-25 represses the expression of F-box-containing and WD repeat domain 7 (FBXW7) and Dickkopf Wnt signaling pathway inhibitor 3 (DKK3), thereby promoting the proliferation and migration of glioma cells (Peng et al., 2019). miR-876-5p plays an important role in the lncHOXC-AS2 (LncRNA HOXC cluster antisense RNA 2)-miR-876-5p-ZEB1 signaling pathway to promote EMT in gliomas (Dong et al., 2019).

In contrast, some miRNAs inhibit invasion and migration by suppressing the EMT process.

Snail 1 is one of the zinc finger transcriptional repressors and its pathological expression is closely associated with the EMT program and tissue invasive activity of cancer cells. After transcription, miR-22 inhibits glioma cell growth by suppressing Snail 1 and induces glioma cell cycle arrest affecting their migration and invasion (Zhang et al., 2020). Conversely, overexpression of miR-382-5P also inhibits the expression of the glioma cell epithelial markers Snail and Slug, as well as possibly negatively regulates the Y box binding protein 1 (YBX1) gene, thereby suppressing glioma migration, invasion, and the EMT process (Wang J. et al., 2019). miR-124 and miR-16 can inhibit the EMT process by suppressing the transcriptional activity of Slug, further suppressing glioma migration and invasion (Xie et al., 2012; Wang Q. et al., 2014). Of course, some related miRNA signaling pathways can also inhibit EMT. miR-451 also reduces invasion, migration, and EMT in glioma cells by targeting CAB39, and thus, inhibiting the Phosphoinositide 3-kinase/protein kinase B/Snail (PI3K/Akt/Snail) signaling pathway (Nan et al., 2021a) miR-661 directly regulates the target gene RAB3D and inhibit AKT/GSK3β signaling, which in turn inhibits glioma cell migration, invasion and EMT (Jin et al., 2020). miR-128 plays a negative regulatory role in the SNAI1/miR-128/SP1 axis, counteracting this role of SNAI1 in promoting glioma progression and inhibiting glioma aggressiveness (Dong et al., 2014). miRNAs can cause downregulation of EMT expression by targeting and regulating their target genes. miR-205 inhibits glioma growth, invasion, and reverses EMT by downregulating its target HOXD9 (Dai et al., 2019).

As mentioned above, it has been shown that a large number of miRNAs play an important role in the EMT process, and therefore, prevention of EMT may be a promising approach to combat invasion and migration.

### miRNAs and hypoxia

Hypoxia plays a driving role in tumor adaptation, promotion of tumor progression, and resistance to therapeutic effects (Valtorta et al., 2020). Hypoxia-inducible factor 1a (HIF-1a) is a major transcriptional regulator of hypoxia-induced gene expression (Pientka et al., 2012).

miR-576-3p overexpression, in turn, decreases the migratory and proangiogenic properties of hypoxia-treated glioma cells *via* suppressing HIF-1 expression (Hu et al., 2019). miR-23b affects the survival and invasion of glioma cells by targeting and modulating the von Hippel–Lindau (VHL), thereby inhibiting the Hypoxia-inducible factor 1a/vascular endothelial growth factor (HIF-1a/VEGF) pathway and b-catenin/Tcf-4 transcription (Chen et al., 2012a). Insulin-like growth factor 1 receptor (IGF-1R) is another major factor in hypoxia. Overexpression of miR-181b is able to target IGF-1R and its downstream signaling pathways PI3K/AKT and the RAS/RAF/MAP kinase-ERK kinase (MEK)/extracellular-signal-regulated kinase (ERK) (MAPK) (MAPK/ERK1/2), thereby inhibiting cell migration, invasion, and tumorigenesis (Shi et al., 2013).

The migration, invasion, and angiogenesis of glioma cells are closely related to hypoxia, and miRNAs targeting their association may become an important part of controlling tumor metastasis.

### miRNAs and angiogenesis

An important basis for solid tumor growth is angiogenesis and regulation by miRNAs (Dai et al., 2018). The angiogenic state is determined by the balance of anti-angiogenic and pro-angiogenic molecules and VEGF is regarded as the most essential angiogenic factor (Rudge et al., 2007; Yancopoulos, 2010). miRNAs are involved in regulating angiogenesis during glioma formation and affecting the aggressiveness of gliomas (Liu et al., 2014; Yao et al., 2014). miR-24 may be involved in glioma angiogenesis through upregulation of vascular endothelial growth factor and TGF-β expression levels as well as intracellular AKT and β-catenin signaling pathways (Dai et al., 2018). Glioma angiogenesis is dependent on the proliferation, migration, and tube formation of human brain microvascular endothelial cells (HBMVEC), and miR-107 overexpression may inhibit HBMVEC by downregulating VEGF expression, thereby suppressing cell invasion and migration (Chen et al., 2016).

Thus, maintaining a balance between pro- and anti-vascularity may help to inhibit cancer cell migration.

### Interaction between miRNAs and the tumors microenvironment

The TME plays an important role in tumor development and therapy and consists of different cell populations, such as immune cells, fibroblasts, tumor-associated macrophages (TAM), endothelial cells, signaling molecules, and extracellular matrix (ECM) components. It is also involved in regulating disease progression (Calabrese et al., 2007; Cheng et al., 2013; Matarredona and Pastor, 2019; Jeanmougin et al., 2020).

Tumor-associated macrophages are particularly important components of TME and are indispensable in the regulation of tumor development and antitumour immune responses (Roesch et al., 2018). In the TME, TAM promotes tumor angiogenesis and immunosuppression by secreting factors associated with stimulation of tumor invasion, survival, and proliferation (Qian and Pollard, 2010). Of these, M1-TAMs exhibit tumor suppression, whereas M2-TAMs exhibit tumor support (Chen et al., 2017). M2-TAMs promote glioma growth and invasion (Hambardzumyan et al., 2016). miR-32 promotes the transformation of M2 macrophages through the PI3K/AKT signaling pathway, thereby enhancing glioma proliferation and migration, mainly because inhibition of THP1 cells affects their internal phosphatase and tensin homolog (PTEN) expression, which in turn negatively regulates the PI3K/AKT signaling pathway (Bao and Li, 2019). In glioblastoma (GBM), M2-TAMs inhibit miR-340-5p by upregulating transforming growth factor β-1 and promoting basement membrane HMGA-2 expression (Liu Y. et al., 2019).

TNF-α is overexpressed and secreted in the TME and is a major regulator of inflammation (Ramaswamy et al., 2019). It can achieve glioma proliferation, migration, and treatment resistance through activation of the NF-kB signaling pathway (Guo et al., 2017; Geeviman et al., 2018). The TNF-α mediated NF-kB signaling pathway can be activated and lead to cell proliferation, invasion, and migration after retinoic acid receptor-related orphan receptor A (RORA) inhibition by miR-18a (Jiang et al., 2020).

Similarly, tumor-associated fibroblasts (CAF) and glioma stem cells (GSCs) show an active role in TME. LNC HOXA transcript antisense RNA myeloid-specific 1 (LNC HOTAIRM1), when directly bound to miR-133b-3p, is able to target and regulate its downstream target TGF-β, which is involved in the regulation of fibroblast transformation (transformation of fibroblasts, t-FBS) malignancy in TME of GSCs remodeling. It is also highly expressed in high-grade gliomas and t-FBS, representing a poor prognosis. The reduction of HOTAIRM1 inhibits the proliferation, invasion, migration, and even tumourigenicity of t-FB (Wang H. et al., 2021). Sarkar et al. (2006) showed that partial expression of matrix metalloproteinase (MMPs) can regulate glioma aggressiveness by affecting the degradation process of extracellular matrix (ECM) components. miR-4262 expression downregulation inhibits glioma proliferation and migration by suppressing the expression of MMP2 and MMP13 (Yang et al., 2020), and miR-34a inhibits glioma cell migration by regulating MMP-9 (Wang X. et al., 2019).

Therefore, clarifying the interactions between miRNAs and TME may be an important strategy to address the metastasis issue.

## miRNAs and their molecular mechanisms in the key glioma signaling pathways

It is well documented that the migration and invasion of gliomas depend on different molecular mechanisms mediated by miRNAs. These miRNAs and their corresponding targets, as well as the corresponding molecular mechanisms, are shown in Table 2.

**Table 2:** The miRNA-related molecular mechanisms of glioma invasion and migration.

**Mechanism**	**miRNAs**	**Type of study**	**Subjects**	**Pression or knock out**	**Cell culture**	**Alteration**	**Downstream**	**References**
Wnt/β-catenin	miR-1825	Clinical	Human	Over pression	BeNa Culture Collection	↓	CDK14	Lu F. et al., 2020
	miR-10a	Clinical	Human	Over pression	DMEM	↑	MTMR3	Yan et al., 2019
RAS	Let-7a	Clinical	Human	Over pression	DMEM	↓	K-RAS	Wang et al., 2013
	miR-143	Clinical	Human	Over pression	DMEM	↓	N-RAS	Wang L. et al., 2014
AKT	miR-92a	Clinical	Human	Over pression	RPMI-1640	↑	RAP18	Liu P. J. et al., 2019
	miR-24	Clinical	Human	Over pression	DMEM	↑	CDX1	Lei et al., 2021
	miR-145	U87-MG, U251n, T98G and HF66 cells	Human	Over pression	RPMI-1640	↓	ADAM17	Zheng et al., 2007; Lu et al., 2013
	miR-221/222	U251 glioblastoma cells and rat C6 glioma cells	Human	Over pression	DMEM	↑	MMP-2, MMP-9	Zhang et al., 2010
TGF-β	miR-132	Clinical	Human	Over pression	DMEM	↑	Smad7	Wang et al., 2015
	miR-663a	Clinical	Human	Over pression	DMEM-F12 medium	↓	KDM2A	Wang L. et al., 2021
NF-KB	miR-182	Clinical	Human	Over pression	DMEM	↑	CYLD	Song et al., 2012
Notch	miR-107	U87, U251 and A172 cells	Human	Over pression	DMEM	↓	Notch2	Chen et al., 2013
	miR-524-5p	CGGA data	Human	Over pression	DMEM	↓	Jagge-1, Hes-1	Chen et al., 2012b
JAK/STAT	miR-133a	Clinical	Human	Over pression	FBS	↓	CTGF	Zhang P. et al., 2019
	miR-221/222	GEO data	Human	Low pression	RPMI-1640	↓	SOCS3	Xu et al., 2019
LncRNA/miRNA/mRNA	miR-128-3P	Clinical	Human	Over pression	DMEM	↑	GREM1	Fu et al., 2018
	miR-138-2-3P	GEO data and clinical	Human	Over pression	DMEM	↓	TRIM24, NCK1-AS1	Huang et al., 2020
DNA promoter methylation	miR-148	Clinical	Human	Over pression	DMEM	↓	DNMT1	Li et al., 2019
	miR-141	U87, U251 and A172	Human	Low pression	DMEM and FBS	↑	SKA2	Bian et al., 2016

CDK14, Cyclin-dependent kinases-14; MTMR3, myotubularin-related protein 3; TFF3, Trefoil Factor 3; PTP, Protein tyrosine phosphatases; PIP3, phosphatidylinositol-3,4,5-trisphosphate; MMP, matrix metalloproteinase; KDM2A, Lysine demethylase 2A; IKK-b, IjBs kinase; CTGF, connective tissue growth factor; SOCS3, suppressor of cytokine signaling-3; PCDH8, protocadherin-8; GREM1, Gremlin1; BMP; bone morphogenetic Protein; YAP, Yes-associated protein; TRIM24, tripartite motif-containing 24; CDX1, caudal-type homeobox 1; ADAM17, A disintegrin and Metalloprotease 17; SKA2, spindle and kinetochore associated complex subunit 2;CGGA, Chinese Glioma Genome Atlas; GEO, Gene Expression Omnibus; FBS, fetal bovine serum; SKA2, spindle and kinetochore associated complex subunit 2.

### miRNAs in the Wnt signaling pathway

The wnt/β-catenin signaling pathway is one of the most important molecular pathways in the development of many human tumors, including gliomas, and it encompasses and is closely associated with the proliferation, migration, and invasion of tumor cells and their angiogenic processes (Wang et al., 2018; Gong et al., 2019; Huo et al., 2020). Upregulation of miR-1825 targets cell cycle protein-dependent kinase-14 (CDK14) *via* the Wnt/β-catenin protein pathway to inhibit invasion and migration (Lu F. et al., 2020). And miR-10a can promote glioma invasion and migration by targeting the 3′ untranslated region of myotubularin-related protein 3 (MTMR3), which regulates the wnt/β-catenin signaling pathway (Yan et al., 2019).

### miRNAs in the EGFR signaling pathway

Epidermal growth factor receptor signaling pathway is one of the multiple oncogenic pathways regulated by miRNAs that control cell proliferation, invasion, migration, angiogenesis, and apoptosis (Xu et al., 2021). Activation of the EGFR signaling pathway also initiates its downstream signaling pathways, including the PI3K (phosphatidyl-inositol-3 Kinase)/ATK (protein kinase B)/mTOR (mammalian target of rapamycin) and Ras (rat sarcoma virus)/Raf (rapidly accelerated fibrosarcoma)/MEK (mitogen-activated protein-kinase)/ERK (extracellular signal-regulated kinase) pathways (Ivliev et al., 2010; Mazzoleni et al., 2010; Motomura et al., 2011). Mutant forms of EGFR associated with glioma exhibit constitutive kinase activity, long-term stimulation of RAS signaling to promote cell cycle progression, and activation of the PI3K/AKT pathway to promote cell proliferation and migration (Rong et al., 2009; Ivliev et al., 2010; Mazzoleni et al., 2010).

#### Ras signaling pathway

The RAS oncogene family includes three members: K-RAS, N-RAS, and H-RAS. Let-7a affects cell proliferation, apoptosis, migration, and invasion by regulating its functional target, K-RAS, and thus, its downstream PI3K/AKT signaling pathway (Wang et al., 2013). miR-143 overexpression inhibits the proliferation, migration, invasion, and angiogenesis process of glioma cells by suppressing its target gene N-RAS; thus, activating the downstream PI3K/AKT signaling pathway (Wang L. et al., 2014).

#### AKT-related signaling pathway

AKT plays an important role as a serine-threonine protein kinase in human malignancies, causing many downstream effects of PI3K, phosphorylating multiple substrates involved in regulating tumorigenesis, and promoting glioma cell survival through activation of mTOR, TSC2, and S6. It also promotes the invasion of glioma cells while increasing the expression of MMPs (Pu et al., 2004; Zhang et al., 2009). Moreover, AKT is not only negatively regulated by PTEN but also activated by several growth factors and their receptors, especially EGFR (Narita et al., 2002; Choe et al., 2003; Golding et al., 2009). AKT in glioblastoma is activated in the presence of EGFR overexpression or mutations. The deletion of PTEN in gliomas is closely associated with AKT activation (Zhang et al., 2010). A study confirmed that the PI3K/AKT signaling pathway might contribute to glioma cell proliferation and invasion by inactivating apoptosis-related signals (Chaudhuri et al., 2018). miR-92a overexpression promotes glioma cell proliferation through the KLF4 (Kruppel-like factor 4)/AKT/mTOR signaling pathway (Liu P. J. et al., 2019). miR-24 promotes glioma development by acting on the caudal-type homeobox 1 (CDX1) target to activate the PI3K/Akt signaling pathway, promote cell proliferation, and induce apoptosis (Lei et al., 2021). A disintegrin and Metalloprotease 17(ADAM17), the target of miR-145, is also a major upstream component of several EGFR precursor ligands (Zheng et al., 2007; Lu et al., 2013), which activate MEK/ERK and PI3K/Akt when EGFR binds to the ligands, leading to aggressive and other malignant phenotypes (Tsatas et al., 2002). MMP-2 and MMP-9 protein expression can be significantly upregulated upon miR-221/222 overexpression and promote proliferation and invasion of glioma cells in the presence of the Akt downstream pathway (Zhang et al., 2010).

### miRNAs in the TGF-β signaling pathway

TGF-β has a dual role, acting as a suppressor in the early stages of tumors growth and displaying a malignant phenotype induced by oncogenic factors during tumors development (Bruna et al., 2007; Yang et al., 2012). The TGF-β signaling pathway is mediated by SMAD proteins (Duan and Chen, 2016). TGF-β is able to promote glioma cell growth through the SMAD and ERK1/2 pathways, thereby activating Nodal expression (Sun et al., 2014). TGF-β concentration and miR-132 levels in glioma cells are positively correlated and promote each other. miR-132 enhances the activation of TGF-β signaling by inhibiting Smad7 expression in glioma cells (Wang et al., 2015). TGF-β also acts as a downstream target of lysine demethylase 2A (KDM2A), which regulates cell proliferation, while upregulation of miR-663a inhibits migration and invasion by suppressing KDM2A (Wang L. et al., 2021).

### miRNAs in the NF-κB signaling pathway

Aberrant activation of NF-κB is observed in many types of human tumors, including gliomas, and is regulated by IJBS kinase (IKK-β), which is involved in cell proliferation, cell migration, and angiogenesis (Goldbrunner et al., 1999; Tektonidis et al., 2011; Yang et al., 2014). miR-451 is able to significantly inhibit the proliferation, invasion, and migration of glioma cells by targeting IKK-β regulated NF-κB (Nan et al., 2021b). A key negative regulator of NF-κB signaling is cylindromatosis (CYLD) deubiquitinase (Kovalenko et al., 2003; Trompouki et al., 2003; Sun, 2010) and miR-182 is able to directly inhibit the ubiquitin binding of CYLD, a component of the NF-κB signaling pathway, thereby inducing an aggressive phenotype in glioma cells (Song et al., 2012).

### miRNAs in the Notch signaling pathway

The Notch signaling pathway is a conserved pathway that controls cell fate and growth evolution (Leong and Karsan, 2006; Sivasankaran et al., 2009; Chen et al., 2012b). In mammals, Notch2 is one of the four members of the Notch receptor family (Notch 1–4) (Wu and Bresnick, 2007) and is involved in the proliferation, invasion, and metastasis of a variety of tumors cells, such as uveal melanoma, gastric cancer, and medulloblastoma (Fan et al., 2004; Boulay et al., 2007; Sivasankaran et al., 2009; Asnaghi et al., 2012; Tseng et al., 2012). Although miR-107 expression is down-regulated in glioma tissues and cell lines, its overexpression can directly target and regulate Notch2, which inhibits the migratory and invasive capacity of glioma cells, a known target for Tenascin-C and COX-2 trans-activation (Chen et al., 2013). miR-524-5p exerts tumors suppression by directly negatively targeting Jagge-1 and Hes-1, two key components of the Notch pathway, which are direct functional targets of miR-524-5p (Chen et al., 2012b).

### miRNAs in JAK/STAT signaling pathway

The activity of Janus kinase (JAK) and signal transducers and activators of transcription (STAT) pathway is a major signaling mechanism for many growth factors and cytokines (Rawlings et al., 2004). It has also been shown that the development of human gliomas is closely related to the JAK/STAT pathway (Tu et al., 2011). miR-133a inhibits connective tissue growth factor (CTGF) expression and JAK/STAT activation, thereby suppressing glioma cell migration and invasion (Zhang P. et al., 2019). The miR-221/222 cluster may affect the angiogenic process in glioblastoma cells by regulating the activation of the JAK/STAT pathway through the regulation of the suppressor of cytokine signaling-3 (SOCS3) (Xu et al., 2019). Oncogene transcription factor signaling and activator of transcription 3 (STAT3), a component of the JAK/STAT3 signaling pathway, is also particularly important in many physiological and pathophysiological processes, such as tumors growth, invasion, and metastasis (Bromberg and Darnell, 2000; Yu and Jove, 2004; Doucette et al., 2012).

### lncRNA/miRNA/mRNA axis

The long noncoding RNAs (lncRNA)/miRNA/mRNA axis pair is indispensable in tumor invasion and metastasis. lncRNA acts as a transcriptional regulator of mRNA by competitively binding miRNA, a process known as the sponge effect. New studies of the lncRNA/miRNA/mRNA regulatory network are important for understanding the mechanisms of tumor invasion and migration. LncRNA PVT1 affects the downstream proteins BMP2 and BMP4, which regulate the bone morphogenetic protein (BMP) signaling pathway, by interacting with miR-128-3p and regulating Gremlin 1(GREM1), thereby affecting glioma cell proliferation, invasion, and migration (Fu et al., 2018). Long non-coding RNA NCK1-AS1 (LncRNA NCK1-AS1) binds to miR-138-2-3P and regulates the indirect target of NCK1-AS1 containing tripartite pattern 24 (TRIM24) to activate its downstream Wnt/β catenin, which in turn affects glioma cell invasion and migration (Huang et al., 2020). Therefore, further studies on non-coding RNAs can reveal new mechanisms that may help us further understand epigenetic modifications and also provide more relevant information on the pathogenesis of human gliomas (Bian et al., 2016).

### miRNAs and methylation

DNA methylation is an important mechanism of miRNA deregulation in tumors. Epigenetic modifications are closely related to gene expression. Transcriptional silencing may lead to the methylation of DNA promoters located at or near CpG islands observed during tumorigenesis, with subsequent manipulation of chromatin structure and gene transcription (Ozsolak et al., 2008). Promoter hypermethylation is thought to be a mechanism for the downregulation of tumor suppressor genes in human cancers (Esteller et al., 2001). DNA methylation is established by DNA methyltransferases, in which DNA (cytosine-5-)-methyltransferase 1 (DNMT1) maintains the methylation pattern and can be downregulated post-transcriptionally by the miR-148 family (Li et al., 2019). DNMT1 mediates promoter methylation, leading to downregulation of miR-141 gene expression, targeting regulation of spindle and kinetochore-associated complex subunit 2 (SKA2), and promoting glioma invasion and metastasis (Bian et al., 2016).

## The clinical value of miRNAs involved in glioma metastasis

### miRNA as a biomarker for the diagnosis and prognosis of glioma

Currently, the diagnosis of glioma is based on imaging, such as computed tomography (CT) and magnetic resonance (MRI) (Kopkova et al., 2019), which are the most widely used tools but are expensive, do not improve early diagnosis (Kreisl et al., 2009), and have limited resolution (Server et al., 2009; Zakaria et al., 2014). Additionally, histological examination is fundamental for the diagnosis and prognosis of gliomas, but this requires access to tumor tissue through invasive neurosurgery (Ma et al., 2018). Given this situation, there is an urgent need for an improved method for the minimally invasive and rapid diagnosis and monitoring of gliomas, and miRNA may be such an ideal biomarker (Wei et al., 2016; Ma et al., 2018). Circulating specific miRNAs, such as plasma or serum, are more stable and can be detected in clinical specimens, showing great potential as convenient and non-invasive biomarkers (Chen et al., 2008; Mitchell et al., 2008). Interestingly, many studies have found that circulating miRNAs are potential diagnostic or prognostic biomarkers for cancer and other disease classifications, including prostate cancer (Selth et al., 2013), breast cancer (McDermott et al., 2014), and gastric cancer (Zhu et al., 2014). Recently, a study explored the feasibility of using aberrant single miRNAs as diagnostic or prognostic biomarkers for glioma, such as miR-205 (Yue et al., 2016), miR-128 (Sun et al., 2015), and miR-210 (Lai et al., 2015). These results suggest that this technology has promising applications.

Studies have confirmed that miRNAs are involved in the pathogenesis of several human cancers (Wang and Chen, 2014) and that they may play a role in biological processes by regulating the expression of key genes at the post-transcriptional level (Deng et al., 2018). A large number of miRNAs can be detected in extracellular fluids, including plasma, serum, urine, cerebrospinal fluid (CSF), saliva, and other human body fluids (Turchinovich et al., 2011). Circulating miR-182 overexpression may be a useful non-invasive biomarker for early diagnosis and prediction of clinical prognosis in gliomas (Xiao et al., 2016). miR-205 in serum was determined to be a new and valuable diagnostic biomarker for glioma and an independent prognostic indicator for overall survival (OS) in advanced tumors (Yue et al., 2016).

Furthermore, in complex exosomes, miRNAs are relevant biomarkers for the diagnosis and prediction of cancer due to their role as regulators of tumors, both as oncogens and carcinogens (Jonas and Izaurralde, 2015; Touat et al., 2015). Abnormal expression of serum exosomal-(exo) miR-210 is positively correlated with glioma grade in patients with glioma (Lan et al., 2020). In contrast, exosomal miR-2276-5p is negatively correlated with tumor grade (Sun et al., 2021). And the expression of serum exosome miR-301a may serve as a new biomarker for the diagnosis of glioma and may predict the prognosis of patients (Lan et al., 2018).

Currently, histopathology is the gold standard for the diagnosis of glioma staging and grading, but the histopathology of tumor specimens obtained by resection or stereotactic biopsy relies heavily on specific structural similarities between tumor cells and non-tumorigenic glial cells (Riemenschneider et al., 2010). Tumor staging and follow-up after glioma treatment rely on neuroimaging, of which MRI is particularly important. For cancer patients, blood biomarkers are highly effective in screening, early diagnosis, detection of efficacy, and predictive review (Sawyers, 2008). Hence, the search for non-invasive objective biomarkers, especially in serum or plasma, is crucial for the diagnosis of glioma.

The development of glioma is closely related to the expression levels of miRNAs and predicts the prognosis of patients. Therefore, detecting the expression levels of miRNAs in patients with glioma may assist with the selection of optimal tumor treatment. Some miRNAs associated with invasion and migration are considered potential markers to predict the prognosis of patients with glioma. Low expression of miR-135a-5p suggests a poor prognosis for patients with glioma (Luo et al., 2019). The expression of miR-497 in patients with glioma is positively correlated with survival time (Feng et al., 2016). Decreased serum levels of miR-376a, miR-376b, and miR-376c in human patients with glioma indicate a poor prognosis (Huang et al., 2017). Since serum or plasma contains more miRNAs of non-tumor origin than CSF, CSF may be considered a more appropriate source of diagnostic tissue. Some reports also suggest that exosomal miRNAs are more likely to be of tumor origin than CSF, and therefore can be used to predict prognosis (Zhu et al., 2012). In addition, tumor-derived exosomes can convert fibroblasts and mesenchymal stem cells into myofibroblasts, thereby promoting tumor angiogenesis and metastasis (Liu et al., 2018). High expression of miR-454-3p in exosomes or low expression in tissues is strongly associated with poor prognosis (Shao et al., 2019).

Although miRNAs theoretically have promising applications as diagnostic or prognostic biomarkers for glioma, no specific miRNAs have been validated for glioma. Second, although fluorescent quantitative real-time PCR (qPCR) is the most commonly used techniques for quantifying miRNA, it still has limitations, such as low sensitivity and poor accuracy in detecting copy templates (Ma et al., 2013). Three-dimensional DNA nanomachine can be combined with toehold-mediated strand displacement reaction for sensitive electrochemical detection of miRNA (Lu H. et al., 2020).

### miRNA-based targeted therapy for the invasion and migration of gliomas

A defined miRNA may have multiple different miRNA targets and a single target may be regulated by multiple miRNAs (Friedman et al., 2008). There is a growing interest in building large and complex regulatory networks for targeted therapies. miRNAs play an important role in both normal and abnormal physiological activities. Recently, a large number of miRNAs have been closely related to cancer; therefore, miRNAs have a promising future as targets for the treatment of different types of cancer (Musilova and Mraz, 2015).

To date, miRNAs have been shown to function as carcinogens (Calin and Croce, 2006) and likewise as tumor suppressors (Calin et al., 2002; Bonci et al., 2008). Therefore, activation of oncogenic factors or inhibition of oncogenic factors is the most basic miRNA therapeutic approach (Chi and Zhou, 2016). Although the envisioned results were not achieved by applying antisense miRNA oligonucleotide constructs (Gentner et al., 2009), by applying double-stranded miRNA mimics (De Guire et al., 2010), or viral/liposome delivery systems (Xie et al., 2019), these were inevitably confounded by toxic effects, including off-target and immune-related effects (Xuan et al., 2015). Fortunately, liposomes coated with polyethylene glycol (PEG) have been used as carriers for delivering miRNA, so that it could penetrate the blood-brain barrier and act on the target (Jonas et al., 2015). This new nanocarrier can wrap miRNA molecules and improve their stability, which helps to obtain higher transduction efficiency and effective bioactivity, promising the clinical application of this efficient and less toxic method of miRNA transfection. The nanocarrier polyethylene glycol methyl ester (mPEG-g-PEI) enhanced the transfection rate of miR-135a by improving the uptake impact of normal glial cells and glioma cells. It can considerably improve glioma patients' survival and prevent tumor development (Liang et al., 2017).

However, miRNA-targeted therapies still face several serious problems, from basic to clinical applications. First, cell membranes are negatively charged, and miRNA molecules cannot interact directly with them, but when miRNAs are wrapped by positively charged PEI, they can facilitate their uptake by cell membranes (Torchilin et al., 2003). Second, non-targeted effects of non-specific miRNAs, such as the development of less toxic miRNA mimics and inhibitors, have also achieved results beyond our expectations, but this requires in-depth studies on pharmacological, pharmacokinetic, and pharmacodynamic factors (Moody et al., 2017; Zhang et al., 2018). Third, the development of an efficient and less toxic gene delivery system is a major challenge for gene therapy. Non-viral vectors are potentially an attractive option, but there remains a need to improve their transfection efficiency and reduce their toxicity (Godbey et al., 1999). Finally, nanoparticle-based delivery systems may be a good application for miRNAs in organisms. Nanoparticles made from synthetic natural cationic polymers or cationic substances as one of the non-viral gene delivery vehicles have the potential to overcome the intracellular and extracellular barriers of the organism, ensuring that miRNAs are not degraded by serum nucleases and also increase uptake by target cells. After uptake, miRNA multimers can then be released from endosomes into the cytoplasm and into their pathways (Liang et al., 2017).

## Discussion

Dysregulation of miRNAs is involved in regulating the aggressive process of gliomas and leads to poor prognosis and treatment resistance. To improve the prognosis of patients, it is imperative to gain insight into the expression of miRNAs to partially control their tumor invasion and metastasis. In this study, we reviewed the progress of research on miRNAs related to glioma invasion and metastasis and discussed their roles. We have summarized the clinical significance (diagnosis, prognosis, and treatment) of some miRNAs in glioma, and have attempted to provide new ideas to further explore the role of miRNAs in glioma.

Aberrant expression of miRNAs plays an important role in the migration and invasion of gliomas. However, uniform standards and protocols are essential for miRNA studies, as the expression patterns may be inconsistent due to variations in criteria and methods between experiments. In addition, comprehensive genomic searches and bioinformatics analyses are necessary to find more miRNAs associated with glioma migration and invasion, thus, improving the accuracy of clinical applications.

Although miRNAs in many extracellular fluids, such as plasma, serum, urine, CSF, and saliva have profound implications in diagnosis or prediction of prognosis, their validity still requires further investigation. Therefore, a highly sensitive, specific, and low-cost method to detect miRNAs must be established. For example, miRNAs can be electrochemically detected when 3D DNA nanomachines are combined with toe-mediated SDR (Lu H. et al., 2020). It is also a promising tool for early screening by detecting miRNA-containing exosomes.

In recent years, miRNAs have emerged as promising targets for the treatment of cancer. miRNAs can regulate several mRNAs simultaneously and are more effective than targeting individual mRNAs (Yang et al., 2017). Recent advances in miRNA direct repair (miRNA mimics) and miRNA inhibition therapies (antisense oligonucleotides, antiglycans, locked nucleic acid anti-miRNA, and small molecule miRNA inhibitors) make miRNAs ideal candidates for entry into clinical trials for glioblastoma. The expression levels of miRNAs that are dysregulated in different diseases may be restored by the action of the aforementioned complexes. Moreover, Recent studies have identified the microbubbles Lipid-polymer hybrid nanoparticles (MBs-LPHNs-CRGD) delivery system as a potentially efficient targeted gene delivery system in GBM (Yang et al., 2021).

## Conclusion

Overall, we reviewed the biological significance and clinical potential of miRNA in glioma, which can guide clinical diagnosis and treatment and provide some theoretical basis for further basic research. Identification of more sensitive miRNA biomarkers for the diagnosis and treatment of glioma is imperative for improving the quality of life and prognosis of patients.

## Author contributions

XG and FM conceived, drafted, supervised, and revised the manuscript. XG collected and prepared the related references. HJ and LC drew the figures. All authors read and approved the final manuscript.

## Conflict of interest

The authors declare that the research was conducted in the absence of any commercial or financial relationships that could be construed as a potential conflict of interest.

## Publisher's note

All claims expressed in this article are solely those of the authors and do not necessarily represent those of their affiliated organizations, or those of the publisher, the editors and the reviewers. Any product that may be evaluated in this article, or claim that may be made by its manufacturer, is not guaranteed or endorsed by the publisher.
